# Development of Nipah virus mRNA vaccine for pandemic preparedness

**DOI:** 10.3389/fimmu.2026.1843559

**Published:** 2026-06-05

**Authors:** Jeong-In Kim, Munazza Fatima, Eun-Hye Seo, Pil-Gu Park, Kee-Jong Hong

**Affiliations:** 1 Department of Bio-Medical Sciences, GAIST, Gachon University, Incheon, Republic of Korea; 2 Department of Microbiology, College of Medicine, Gachon University, Incheon, Republic of Korea; 3 Lee Gil Ya Cancer and Diabetes Institute, Gachon University, Incheon, Republic of Korea; 4 Department of Life Science, College of BioNano Technology, Gachon University, Seongnam, Republic of Korea

**Keywords:** mRNA, Nipah virus, pandemic, pseudovirus, vaccine

## Abstract

**Introduction:**

Nipah virus (NiV) is a highly pathogenic zoonotic virus associated with a high case fatality rate and human-to-human transmission, and it is listed as a priority pathogen by the World Health Organization (WHO) due to the lack of approved therapeutics or vaccines. NiV is classified as a biosafety level 4 (BSL-4) pathogen, which severely limits experimental studies in the absence of specialized containment facilities. Therefore, the establishment of pseudovirus-based surrogate models is essential for NiV vaccine research.

**Methods:**

In this study, single-antigen mRNA–lipid nanoparticle (mRNA-LNP) vaccines encoding the Nipah virus fusion protein (NiV-F) and attachment protein (NiV-G) were designed and evaluated using a co-administration strategy of independently formulated single-antigen mRNA-LNPs. A Vesicular stomatitis virus (VSV)-based NiV pseudovirus system was established and applied to assess neutralizing antibody responses in a mouse model.

**Results:**

The synthesized mRNA-LNP vaccines exhibited high purity and relatively consistent physicochemical properties, indicating uniform formulation characteristics. The VSV-based NiV pseudovirus showed a robust infection signal compared to background controls, and optimization of the purification process effectively reduced non-specific background, enabling reliable evaluation of *in vitro* neutralizing activity induced by vaccination. Coadministration of the NiV-F and NiV-G mRNA-LNP vaccines induced detectable antigen-binding antibody responses against both NiV-F and NiV-G antigens. Consistently, pseudovirus-based neutralization assays (PBNA) demonstrated detectable neutralizing activity in sera collected at week 4 post-immunization.

**Discussion:**

This study provides a foundation for future NiV mRNA-LNP vaccine development and presents a flexible vaccine design approach based on co-administration of independently formulated single-antigen mRNA-LNPs in which mixed administration of single-antigen mRNA-LNPs provides a flexible framework for independently modulating antigen composition and enabling antigen-specific immune responses. These findings provide a basis for future studies exploring diverse antigen combinations in NiV vaccine research.

## 1 Introduction

Nipah virus (NiV) is a highly pathogenic zoonotic virus belonging to the family *Paramyxoviridae* and the genus *Henipavirus*. It is an enveloped, single-stranded negative-sense RNA virus classified as a biosafety level 4 (BSL-4) pathogen with fruit bats serving as its natural reservoir and humans as incidental hosts (1, 2). Since its first identification during an outbreak in Malaysia in 1999, sporadic outbreaks have been reported almost annually in Bangladesh and India (3), with multiple documented cases of human-to-human transmission (4). Human NiV infection causes severe neurological and respiratory disease, ranging from febrile illness to acute encephalitis, and is associated with a reported case fatality rate of approximately 60–70% (2, 5). Owing to its high pathogenicity, recurrent community outbreaks, and the lack of approved vaccines or therapeutics, the World Health Organization (WHO) has designated NiV as a priority pathogen (6, 7).

Despite sustained global efforts to develop vaccines against NiV, no licensed vaccine is currently available. Vaccine candidates that have progressed to clinical evaluation have been developed using diverse platforms, including protein subunit vaccines (8), viral vector vaccines (9), and mRNA vaccines (10). Among these, mRNA vaccine platforms have emerged as a particularly attractive approach for addressing high-risk emerging pathogens, owing to their rapid design, scalability, and platform versatility (11). These features make mRNA vaccines well suited for pathogens such as NiV, for which traditional vaccine development is constrained by biosafety requirements.

The primary antigenic targets for NiV vaccine development are the Nipah virus fusion F protein (NiV-F) and the Nipah virus attachment G protein (NiV-G) (12). The NiV-G mediates viral attachment through binding to the host cell receptors ephrin-B2 and ephrin-B3 and is known to be highly antigenic. The NiV-F facilitates membrane fusion during viral entry and exhibits its highest immunogenicity in the pre-fusion conformation (13). Accordingly, recent NiV vaccine studies have focused on antigen design strategies incorporating pre-fusion stabilized NiV-F and NiV-G.

Experimental evaluation of NiV vaccines is further complicated by the classification of NiV as a BSL-4 pathogen, which precludes direct infection studies in laboratories lacking high-containment facilities. To address these limitations, pseudovirus-based infection models have become an essential tool for NiV research, enabling the assessment of viral entry, neutralization activity, and vaccine efficacy without handling the live, highly pathogenic virus. In particular, vesicular stomatitis virus (VSV)-based pseudovirus systems are widely used due to their high expression efficiency and reproducibility across diverse viral glycoproteins (14).

In this study, we investigated an mRNA vaccine strategy based on the co-administration of single-antigen mRNA–lipid nanoparticles (mRNA-LNP) encoding the NiV-F and-G proteins. We hypothesized that mRNA-LNPs encoding NiV-F and NiV-G would elicit antigen-specific humoral responses and generate neutralizing antibodies. Finally, we evaluated a VSV-based NiV pseudovirus system as a surrogate model for assessing *in vitro* neutralizing activity induced by NiV mRNA vaccination in the absence of BSL-4 containment. Unlike previous approaches that integrate multiple antigens into a single construct, this study explores a co-administration strategy using independently formulated single-antigen mRNA-LNPs. Collectively, this study aims to establish an experimental framework for NiV vaccine research under BSL-4–restricted conditions and to explore the feasibility of a vaccination strategy based on the combined administration of single-antigen mRNA-LNPs. This approach provides a flexible framework for independently modulating antigen composition and enabling antigen-specific immune responses under co-administration conditions, supporting the development of combination-based mRNA vaccine strategies.

## 2 Materials and methods

### 2.1 Preparation of cells

HEK293FT and Vero cells (Korean Cell Line Bank, Seoul, Korea) were cultured in DMEM (Cytiva, Washington, USA) and RPMI (Cytiva, Washington, USA) containing 10% fetal bovine serum (FBS, Cytiva, Washington, USA) and 1% antibiotic–antimycotic (Gibco, Massachusetts, USA). Cells were cultured at 37 °C in a 5% CO_2_ atmosphere.

### 2.2 mRNA construct design and *in vitro* transcription and purification

NiV-F and NiV-G gene sequences were obtained from the Nipah virus Malaysia strain (NCBI GenBank accession: UMMC1, AY029767.1). To stabilize the prefusion conformation of NiV-F, four mutations (I114C, L104C, S191P, and L172F) were introduced. For trimer formation, a GCN4 trimerization domain was fused to the C-terminus of NiV-F, and a foldon (Fd) domain was added to the C-terminus of NiV-G (13). Both antigens were codon-optimized for expression in mice. The designed coding sequences (CDSs) were cloned into the CUK3–1 plasmid vector (provided by The Catholic University of Korea, Gyeonggi, Korea), which contains a T7 promoter, 5′ and 3′ untranslated regions (UTRs), and an approximately 100-nucleotide poly(A) tail. To enable CleanCap^®^ (TriLink, California, USA) incorporation during *in vitro* transcription (IVT), the CUK3–1 vector was further modified by site-directed mutagenesis. IVT was performed using the HiScribe^®^ T7 High Yield RNA Synthesis Kit (New England Biolabs, Massachusetts, USA) at 37 °C for 2 h, and no modified nucleotides were incorporated into the synthesized mRNA. The transcribed mRNA was first purified by lithium chloride (LiCl) precipitation and subsequently subjected to a second purification step using the Min-Immune Gold dsRNA Removal Kit (CellScript, Wisconsin, USA) according to the manufacturer’s protocol.

### 2.3 Dot blot

Double-stranded RNA (dsRNA) contamination in purified mRNA samples was evaluated using a dot blot assay. Briefly, 500 ng of purified mRNA was spotted onto a membrane and allowed to air-dry. The membrane was then blocked with 5% skim milk in TBST for 1h. After blocking, membrane was incubated with J2 anti-dsRNA antibody (Cell signaling Technology, Massachusetts, USA) diluted 1:1000 in 1% skim milk in TBST for 1h at room temperature (RT). HRP-conjugated anti-mouse IgG (SouthernBiotech, Alabama, USA) diluted 1:3000 in 1% skim milk in TBST was used as the secondary antibody and incubated at RT for 1 h. Signals were detected using an Enhanced Chemiluminescence (ECL) substrate (BIOMAX, Gyeonggi, Korea). Poly I:C (Invitrogen, Massachusetts, USA) was used as a positive control, and signal intensity was quantified using the ImageJ software.

### 2.4 LNP formulation

NiV-F and NiV-G mRNA-LNP were prepared according to a previously established protocol (15). Briefly, mRNA was prepared in multiples of 120 µg, corresponding to the amount used per formulation. The mRNA was diluted in citrate buffer (pH 4) to generate an mRNA solution at a concentration of 120 µg per 1.5 mL. The lipid mixture was prepared by mixing SM-102 (MedChemExpress, New Jersey, USA), DSPC (Avanti Polar Lipids, Alabama, USA), cholesterol (Avanti Polar Lipids, Alabama, USA), and DEG-PEG2000 (Avanti Polar Lipids, Alabama, USA) in ethanol at a molar ratio of 9.2:2:3.8:1 and dissolved by sonication at 25 °C for 5–10 min. The mRNA solution and lipid mixture were then combined at a volume ratio of 3:1. LNP formulation was performed using the EnCELL Master system (ENPARTICLE, Busan, Korea). The formulated LNPs were transferred to Amicon Ultra-15 centrifugal filter units (Merck Millipore, Massachusetts, USA) and subjected to centrifugation at 3000 g for 20 min to perform buffer exchange into phosphate-buffered saline (PBS). Particle size and polydispersity index (PDI) were subsequently measured by dynamic light scattering (DLS).

### 2.5 Antigen protein expression

HEK293FT cells were seeded into plates to reach 70–80% confluency. NiV-F or NiV-G mRNA was diluted in Opti-MEM (Gibco, Massachusetts, USA), and Lipofectamine 2000 (Thermo Fisher Scientific, Massachusetts, USA) was added at 2.5 times the amount of mRNA. After transfection, cells were incubated for 24 h, the supernatant was removed and proteins were extracted. Protein expression was analyzed by Western blotting. Proteins were separated on 12% SDS–PAGE gels and transferred onto membranes. Membranes were blocked with 5% skim milk in TBST for 1 h. Membranes were then incubated overnight at 4 °C with either NiV-F antibody or NiV-G antibody (Creative Diagnostics, New York, USA) diluted 1:1000 in 5% BSA in TBST. HRP-conjugated anti-mouse IgG (SouthernBiotech, Alabama, USA) diluted 1:10,000 in 5% skim milk in TBST was used as the secondary antibody and incubated at room temperature for 1 h. Signals were detected using an Enhanced Chemiluminescence (ECL) substrate (BIOMAX, Gyeonggi, Korea).

### 2.6 Production of Nipah pseudovirus

Lentivirus-based Nipah pseudovirus was generated as previously described (14). Briefly, HEK293FT cells were seeded in 10 cm dishes to reach 70–80% confluency on the following day. NiV-F and NiV-G sequences were synthesized and cloned into the pcDNA3.1 expression vector (VectorBuilder, Illinois, USA). A total of 22 µg of plasmid DNA was prepared by mixing pcDNA3.1-NiV-F (2 µg), pcDNA3.1-NiV-G (4 µg), Gag/Pol plasmid (6 µg; pMDLg/pRRE, Addgene, Massachusetts, USA), Rev plasmid (2 µg; pRSV-Rev, Addgene, Massachusetts, USA), and a mammalian gene expression lentiviral vector (8 µg; pLV[Exp]-EGFP/Puro-EF1A>Luciferase, VectorBuilder, Illinois, USA) at a ratio of 1:2:3:1:4. Transfection was performed using Lipofectamine 2000 (Thermo Fisher Scientific, Massachusetts, USA). At 5 h post-transfection, the medium was replaced with 10 mL of DMEM-5% FBS. At 48 h post-transfection, culture supernatants were collected and centrifuged at 450 g for 15 min. The clarified supernatants were collected using a 0.45 µm syringe filter. VSV-based Nipah pseudovirus was generated as previously described (16). HEK293FT cells were seeded in 10 cm dishes to reach 70–80% confluency. Cells were transfected with pcDNA3.1-NiV-F (4 μg) and pcDNA3.1-NiV-G (8 μg) at a 1:2 ratio, for a total of 12 μg of plasmid DNA, using Lipofectamine 2000 (Thermo Fisher Scientific, Massachusetts, USA). At 5 h post-transfection, the medium was replaced with 10 mL of DMEM-5% FBS. At 24 h post-transfection, the culture supernatant was removed, and cells were infected with pseudotyped *Δ*G-luciferase recombinant VSV (G*ΔG-luciferase-rVSV; *Δ*G-VSV-Luc; Kerafast, North Carolina, USA) at a multiplicity of infection (MOI) of 0.5 in 4 mL of serum-free DMEM. After incubation for 2 h, the virus inoculum was removed, and cells were washed 3–4 times with PBS to reduce background infectivity. Cells were then incubated for 24 h in DMEM-5% FBS supplemented with anti–VSV-G antibody (Kerafast, North Carolina, USA) diluted 1:1000 to further suppress residual non-specific infectivity. At 24 h post-infection, culture supernatants were collected and centrifuged at 450 g for 15 min. The clarified supernatants were collected using 0.45 µm syringe filter.

### 2.7 Titration of Nipah pseudovirus

Vero cells were seeded into 96-well plates to reach 70–80% confluency on the following day. VSV-based NiV pseudovirus was serially diluted 10-fold from 10¹ to 10¹¹ in serum-free RPMI in a separate plate. The culture medium was removed from the Vero cell–seeded 96-well plates, and 50 µL of each virus dilution was transferred to the corresponding wells. After incubation for 2 h, 50 µL of RPMI-5% FBS was added to each well, and incubated at 37 °C under 5% CO_2_ for 24 h. At 24 h post-infection, luminescence was measured using a One-Glo luciferase assay reagent (Promega, Wisconsin, USA) according to the manufacturer’s instructions using a luminometer (Victor Nivo Multimode Plate Reader, BMG Labtech, Ortenberg, Germany). The 50% tissue culture infectious dose (TCID_50_) was calculated using the Reed–Muench method (17).

### 2.8 Animal immunization

6-week-old female BALB/c mice were purchased from Orient Bio (Gyeonggi, Korea) and maintained under specific pathogen-free (SPF) conditions at the Center of Animal Care and Use (CACU, Incheon, Korea). For the mRNA vaccine group, NiV-F mRNA-LNP (10 µg) and NiV-G mRNA-LNP (10 µg) were mixed in PBS to a total volume of 100 µL and administered intramuscularly (IM) into the thigh muscle of the hind limb at weeks 0 and 2, for a total of two immunizations (13). For the plant-based recombinant protein vaccine (provided by the Korea Research Institute of Bioscience and Biotechnology, Daejeon, Korea), 10 µg of F+G proteins was mixed with 700 µg of aluminum hydroxide (InvivoGen, California, USA) in PBS at a 3:7 ratio and administered via the same route following the same immunization schedule. Blood was collected at weeks 0, 2, and 4 by retro-orbital bleeding under anesthesia with 1–5% isoflurane (Ifran^®^, Hana Pharm, Seoul, Korea) in oxygen and serum was isolated by centrifugation at 1,000 g for 10 min. Animal experiments were conducted with approval from the Center of Animal Care and Use of the Lee Gil Ya Cancer and Diabetes Institute of Gachon University (Incheon, Korea, LCDI-2023-0144).

### 2.9 B cell response assay using ELISA

NiV-F or NiV-G proteins (provided by the Korea Research Institute of Bioscience and Biotechnology, Daejeon, Korea) were coated onto 96-well ELISA plates (Greiner Bio-One, Kremsmünster, Austria) and incubated at 37 °C under 5% CO_2_ for 24 h. On the following day, plates were blocked and immunized mouse sera were added. For the mRNA vaccine group, mouse sera were diluted 1:500 in 1% BSA in PBS, whereas sera from the recombinant protein vaccine group were diluted 1:2500. Plates were incubated at 37 °C under 5% CO_2_ for 3 h. After incubation with primary antibodies, HRP-conjugated anti-mouse IgG (SouthernBiotech, Alabama, USA) diluted 1:2000 in 1% BSA in PBS was added and incubated at 37 °C under 5% CO_2_ for 1 h. Following incubation, TMB substrate (GenDEPOT, Texas, USA) was added and incubated for 15 min at room temperature in the dark. Absorbance was measured at 450 nm.

### 2.10 Pseudovirus-based neutralization assay

PBNA was performed as previously described (16). Briefly, Vero cells were seeded into 96-well plates to reach 70–80% confluency on the following day. Mouse sera were serially diluted 3-fold starting at 1:20 and ending at 1:43,740 in serum-free RPMI. VSV-based NiV pseudovirus was diluted in serum-free RPMI to a concentration of 200 TCID_50_ and incubated with diluted sera at 50 µL per well on ice for 1 h. After removal of the culture medium, 100 µL of the virus–serum mixtures were added to the cells and incubated at 37 °C under 5% CO_2_ for 24 h. Luciferase activity was measured using a luminometer, and relative luminescence units (RLU) were recorded. The percentage of neutralization was calculated using the following equations.


$$100-\left(\frac{RLU\left(sample\right)}{RLU\left(Peudovirus\;only\right)}X\;100\right)$$

Neutralization Antibody Titer 50% (NT_50_) was calculated using GraphPad Prism.

### 2.11 Statistical analysis

All statistical analyses were performed using GraphPad Prism version 8.0.2. A p value of less than 0.05 was considered statistically significant (*P< 0.05; **P< 0.01; ***P< 0.001; ****P< 0.0001; compared with the control group). All data are presented as mean ± SD.

### 2.12 Use of AI-assisted language editing

ChatGPT (OpenAI, GPT-5.2) was used for English language translation.

## 3 Results

### 3.1 Preparation of mRNA vaccine

**Figure 1:**
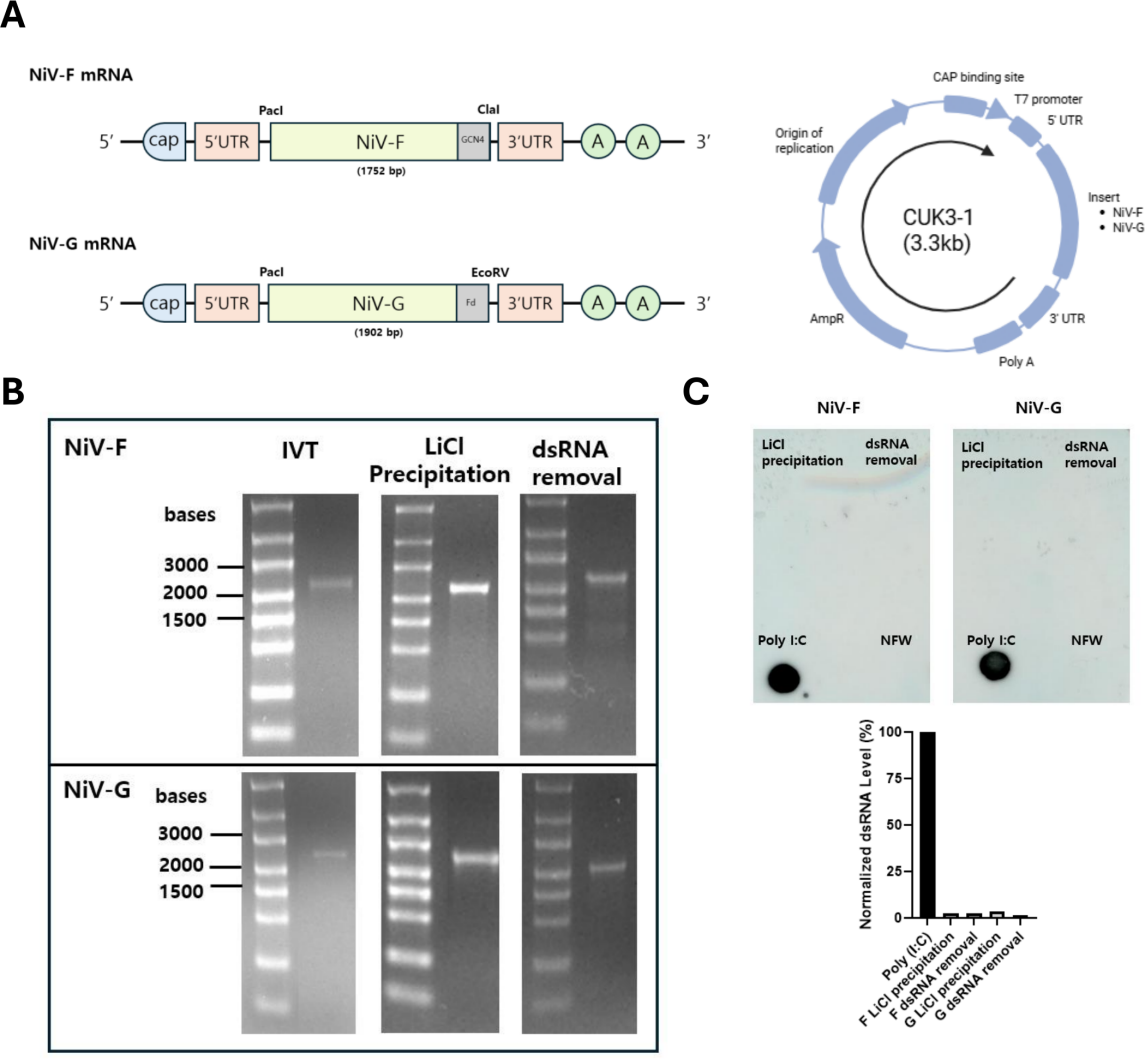
Design of NiV-F and NiV-G mRNA vaccine constructs and evaluation of integrity and dsRNA contamination of NiV-F and NiV-G mRNAs. **(A)** Schematic representation of the NiV-F and NiV-G mRNA constructs and the CUK3–1 vector map used in IVT. Each mRNA construct consists of a 5′ cap, 5′ UTR, a mouse codon-optimized CDS encoding NiV-F (1752 bp) or NiV-G (1902 bp), a C-terminally fused trimerization domain, a 3′ UTR, and a poly(A) tail. The IVT vector contains a T7 promoter, cap-binding site, 5′ and 3′ UTRs, and a poly(A) tail, with the NiV-F or NiV-G CDS inserted between the 5′ and 3′ UTRs. **(B)** Assessment of the integrity and purity of NiV-F and NiV-G mRNA. Samples collected after IVT, LiCl precipitation, and after dsRNA removal were analyzed by electrophoresis on a 1.2% formaldehyde agarose gel. **(C)** Evaluation of residual dsRNA by dot blot analysis using an anti-dsRNA monoclonal antibody (J2 clone). Dot blot signals were quantified using ImageJ software, and dsRNA levels were normalized and expressed as percentages. All experiments were performed in triplicate.

To generate mRNA vaccines, NiV-F and NiV-G of the Nipah virus Malaysia strain were synthesized as individual antigens. For the NiV-F antigen, four stabilizing mutations were introduced to maintain the prefusion conformation. In addition, trimerization domains were fused to each antigen to promote trimer formation, specifically the GCN4 domain linked to NiV-F and the foldon trimerization domain linked to NiV-G (10, 13). (**Figure 1A**). To enhance expression efficiency in the mouse model, the coding sequences were codon-optimized for mouse expression, and the optimized CDSs were cloned into the IVT vector CUK3-1 (**Figure 1A**). IVT of the NiV-F and NiV-G antigens were subsequently performed, and agarose gel electrophoresis was used to verify successful transcription. Electrophoretic analysis showed that the NiV-F mRNA and NiV-G mRNA were approximately 2.1 kb and 2.3 kb in length, respectively, consistent with their expected transcript sizes, suggesting successful IVT of both antigens (**Figure 1B**).

Following IVT, LiCl precipitation and dsRNA removal were performed sequentially for mRNA purification. No smear-like impurities were observed after purification (**Figure 1B**). To further assess dsRNA contamination, dot blot analysis was conducted using an anti-dsRNA antibody. Compared with the positive control (poly I:C), little to no signal was detected in either the LiCl-precipitated samples or the dsRNA-depleted NiV-F and NiV-G mRNA samples, indicating effective removal of dsRNA (**Figure 1C**). Quantitative analysis of the dot blot results using ImageJ further demonstrated that residual dsRNA levels were extremely low relative to poly I:C. These results suggested that the synthesized mRNA-LNP vaccines exhibited high purity and low residual dsRNA levels following purification.

**Figure 2:**
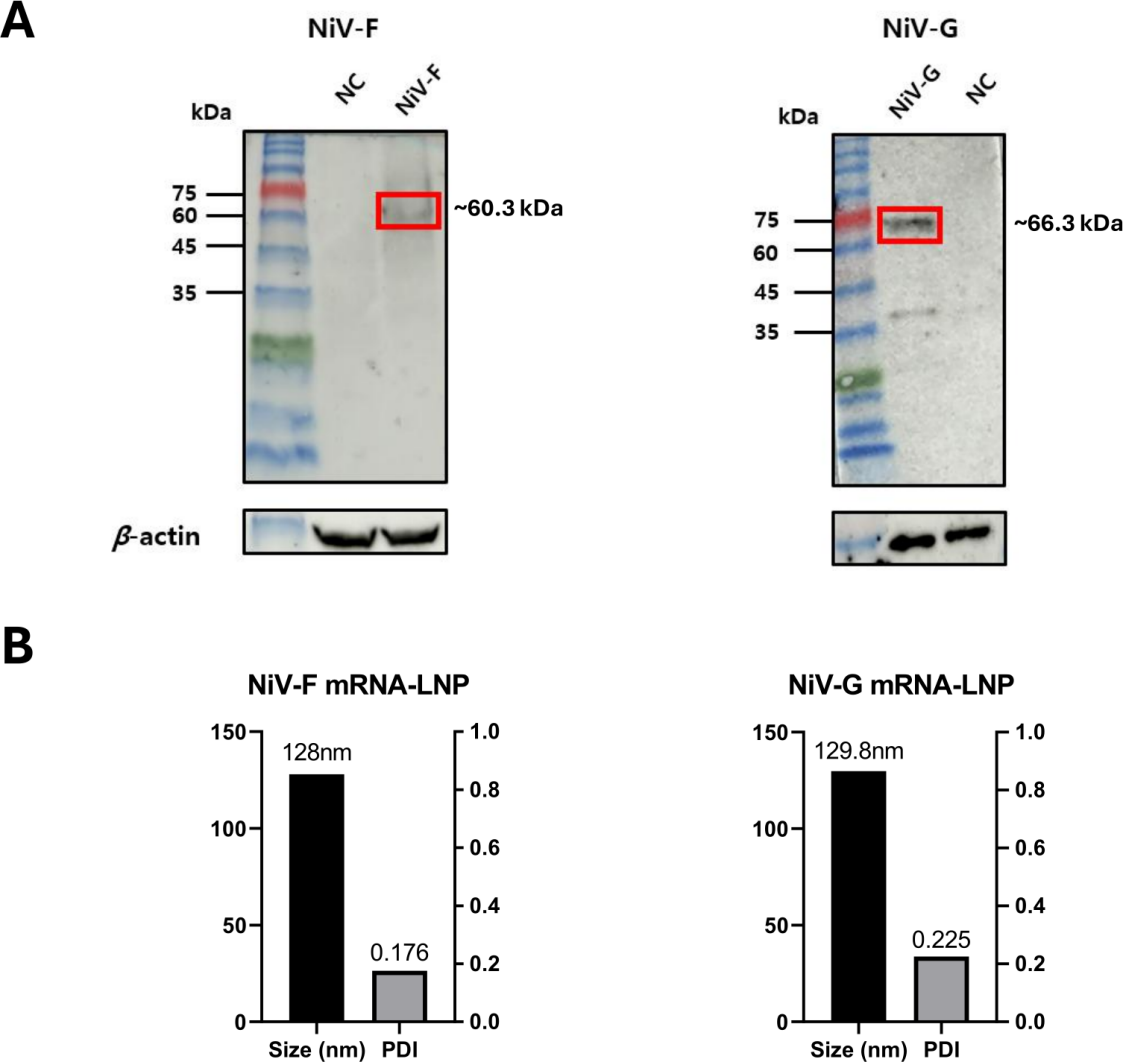
Antigen protein expression and physicochemical characterization of NiV-F and NiV-G mRNA-LNPs. **(A)** Protein expression of NiV-F and NiV-G following transfection of HEK293FT cells with mRNA. Total protein was harvested 24 h post-transfection, quantified, and protein sample was resolved by SDS–PAGE and analyzed by Western blotting. Protein bands were visualized using a ChemiDoc imaging system. Distinct bands corresponding to the expected molecular weights of NiV-F (~60.3 kDa) and NiV-G (~66.3 kDa) were detected by Western blot analysis. **(B)** Particle size and PDI of NiV-F and NiV-G mRNA-LNPs were measured by DLS.

To evaluate antigen expression from the synthesized mRNA, HEK293FT cells were transfected with NiV-F or NiV-G mRNA, followed by Western blot analysis (**Figure 2A**). Although overall signal intensity was relatively weak, distinct bands were observed at the expected molecular weights of 60.3 kDa for NiV-F and 66.3 kDa for NiV-G. These bands were reproducibly detected at the expected molecular weights across independent experiments, suggesting qualitative expression of both antigens. To characterize the physicochemical properties of LNPs encapsulating NiV-F and NiV-G mRNA, DLS analysis was performed (**Figure 2B**). DLS analysis showed that the mean particle size of NiV-F mRNA-LNPs was 129.8 nm (PDI 0.225), and NiV-G mRNA-LNPs also exhibited a similar mean particle size of 128 nm (PDI 0.176), indicating relatively uniform particle size distribution characteristics of the formulated LNPs (15).

### 3.2 Preparation of Nipah pseudovirus

**Figure 3:**
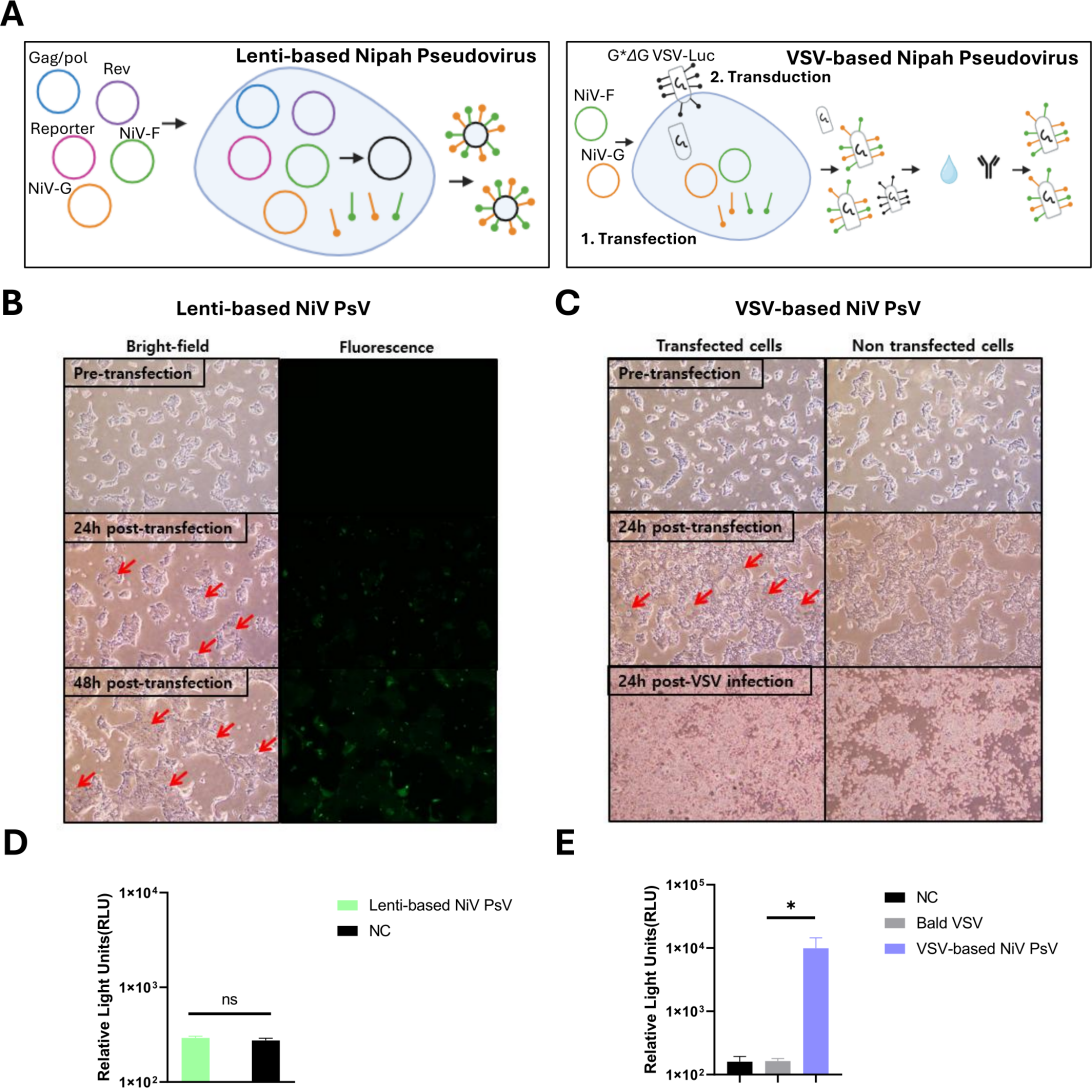
Production of NiV pseudovirus using the established pseudovirus system. **(A)** Schematic workflow of lentivirus-based and VSV-based Nipah virus pseudovirus production. **(B)** Confirmation of protein expression during lentivirus-based Nipah pseudovirus production. HEK293FT cells were transfected with plasmids required for lentivirus-based Nipah pseudovirus production, and protein expression was examined at 24 h and 48 h post-transfection using bright-field microscopy (left) and fluorescence microscopy (right). **(C)** Observation of cellular changes during VSV-based Nipah pseudovirus production. HEK293FT cells expressing NiV-F and NiV-G (left) and cells without expression of any viral proteins (right) are shown. Cells were infected with *Δ*G-VSV-Luc, and cellular morphology was examined 24 h post-infection. **(D)** Evaluation of infectivity of lentivirus-based Nipah pseudovirus. The produced lentivirus-based Nipah pseudovirus was used to infect Vero cells, and infectivity was assessed by measuring RLU using a luciferase assay at 24 h post-infection. Experiments were performed twice, and statistical analysis was conducted using an unpaired t-test (P ≥ 0.05). **(E)** Evaluation of infectivity of VSV-based Nipah pseudovirus. Vero cells were infected with the produced VSV-based Nipah pseudovirus or Bald VSV, and infectivity was assessed by measuring RLU values. Experiments were performed in triplicate, and statistical analysis was conducted using an unpaired t-test (*P< 0.05).

We attempted to generate NiV pseudovirus using lentivirus and VSV-based system. During lentivirus-based production of NiV pseudovirus (**Figure 3A**), syncytium formation mediated by NiV-F was observed at 24-48h post-transfection, and GFP expression from the lentiviral backbone was confirmed (**Figure 3B**). However, when the harvested virus was used for infection, the signal was comparable to background levels, indicating that infectious pseudovirus was not successfully produced (**Figure 3D**).

In contrast, when NiV pseudovirus was generated using a VSV-based system (**Figure 3A**), NiV-F–mediated syncytium formation was observed at 24 h post-transfection (**Figure 3C**). In infectivity assays, the harvested virus showed a signal of approximately 10^4^ RLU, confirming the production of infectious pseudovirus. However, we observed unexpectedly high levels of residual non-specific infectivity in supernatants collected from cells infected with *ΔG*-VSV-Luc in the absence of NiV envelope protein expression (Bald-VSV) when used without purification. Therefore, purification conditions were established, and when the optimized purification protocol was applied and evaluated in comparison with Bald-VSV, non-specific infectivity was effectively suppressed to background levels. As a result, only infectious NiV pseudovirus was selectively retained, and ultimately, purified NiV pseudovirus was successfully obtained (**Figure 3E**).

### 3.3 Co-administration of independently formulated NiV-F and NiV-G mRNA-LNPs induces antigen-specific humoral immune responses

**Figure 4:**
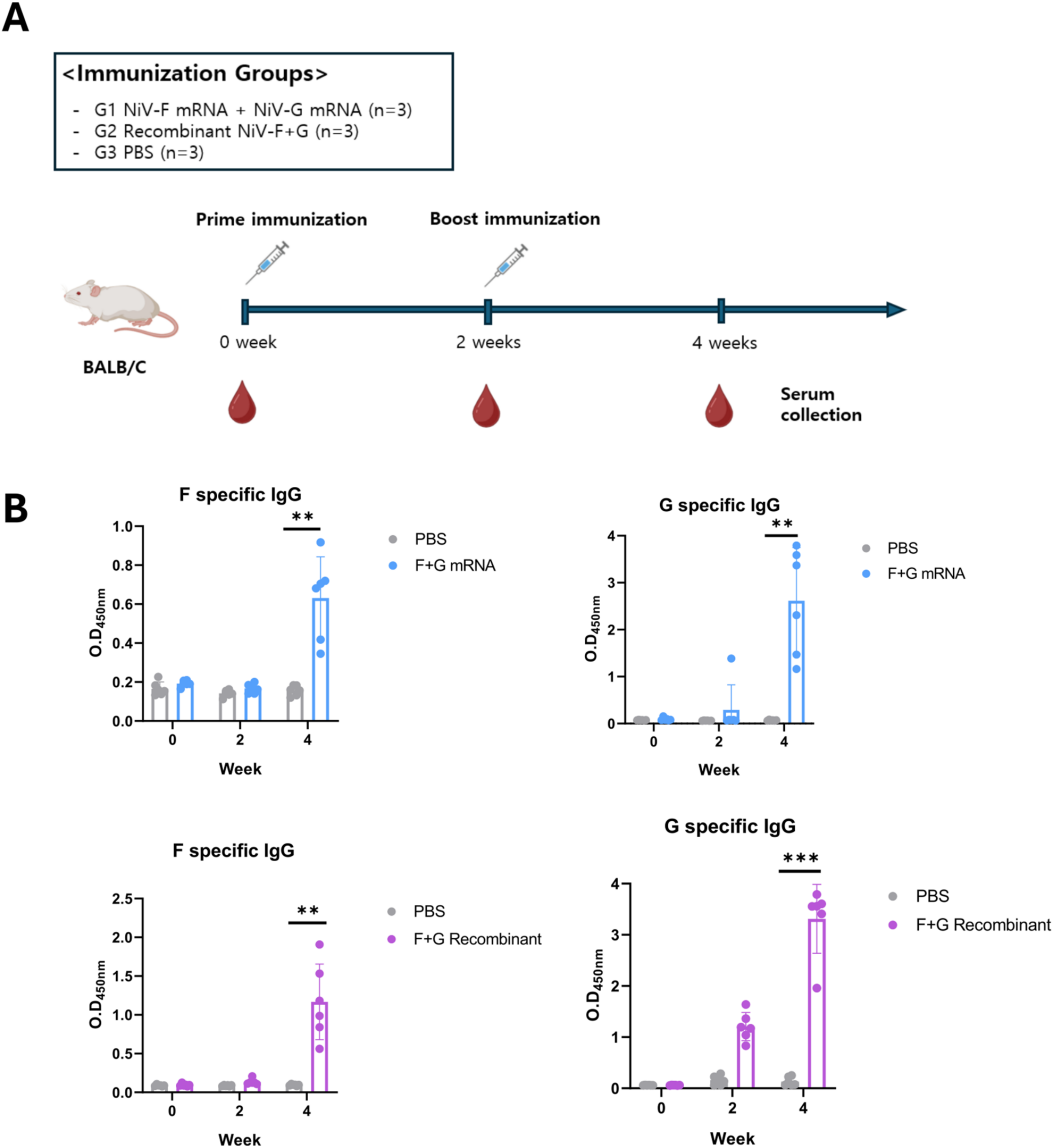
Immunization schedule and antigen-specific IgG responses induced by the NiV mRNA-LNP vaccine. **(A)** Experimental design for evaluation of the immunogenicity of the NiV mRNA-LNP vaccine. Three experimental groups were established: G1, mRNA-LNP vaccine group; G2, recombinant protein vaccine group; and G3, PBS control group. Six-week-old BALB/c mice were immunized twice intramuscularly at weeks 0 and 2, and serum was collected according to the predefined schedule. Each experiment was independently performed using three mice per group under the same experimental conditions. Similar trends were observed across the two independent experiments, and data from individual mice were pooled and analyzed as biological replicates. **(B)** Antigen-specific binding antibody responses against NiV-F and NiV-G were assessed by IgG ELISA using sera collected at weeks 0, 2, and 4 from the mRNA-LNP vaccine group, recombinant F+G protein vaccine group, and PBS control group. The upper panels show NiV-F– and NiV-G–specific IgG responses in the mRNA-LNP–vaccinated group, while the lower panels show antibody responses in the recombinant protein vaccine group. Statistical analysis was conducted using two-way ANOVA (**P< 0.01 and ***P< 0.001).

To evaluate the immunogenicity of the co-administered NiV mRNA-LNP vaccines, three experimental groups were established: a group receiving co-administration of NiV-F and NiV-G mRNA-LNPs, a group receiving a plant-based recombinant NiV F+G protein vaccine used in previous studies, and a PBS control group. The co-administration group receiving NiV-F and NiV-G mRNA-LNPs in combination was immunized intramuscularly with a mixture of NiV-F mRNA-LNP (10 μg) and NiV-G mRNA-LNP (10 μg) at a 1:1 ratio and was designated as group G1. The second group, designated G2, was immunized with the plant-based recombinant NiV F+G protein vaccine consisting of 10 μg of protein mixed with 700 μg of alum as an adjuvant. The PBS-treated group was designated as G3. All groups were immunized twice in weeks 0 and 2. Serum samples were collected at week 0 (pre-immunization), week 2 (pre-boost), and week 4. (**Figure 4A**).

To assess vaccine-induced binding antibody responses, IgG ELISA specific for NiV-F and NiV-G glycoproteins were performed (**Figure 4B**). Sera collected at week 0 and sera from the PBS control group at all time points exhibited uniformly low absorbance values (O.D.< 0.2) across all experimental groups, indicating the absence of antigen-specific antibodies. During week 2 post-immunization, distinct response patterns were observed depending on the antigen. NiV-F specific IgG levels in both the mRNA-LNP group and the recombinant protein vaccine group remained comparable to those of the PBS group, with no marked increase in antibody responses. In contrast, in the recombinant protein vaccine group, NiV-G specific IgG levels began to increase at 2 weeks post-immunization, with all animals exhibiting antibody responses (O.D. 1.2 ± 0.2), whereas in the mRNA-LNP group, antibody responses were observed only in a subset of animals at 2 weeks post-immunization. At week 4 post-immunization, both the mRNA-LNP group and the recombinant protein vaccine group showed pronounced increases in NiV-F and NiV-G specific antibody responses. Notably, under co-administration conditions, antibody responses against NiV-G appeared to be more strongly induced than those against NiV-F. In the mRNA-LNP group, NiV-G specific IgG levels reached an O.D. value of 2.6 ± 1.1, higher than the NiV-F specific IgG levels under the co-administration condition (O.D. 0.6 ± 0.2). Similarly, in the recombinant protein vaccine group, NiV-G specific IgG levels (O.D. 3.3 ± 0.6) were approximately threefold higher than NiV-F specific IgG levels (O.D. 1.1 ± 0.4). Overall, antigen-specific IgG responses were detected in both the mRNA-LNP vaccine group and the recombinant protein vaccine group. Our findings confirmed that the NiV-F and NiV-G antigens used in this study retained their capacity to elicit antigen-specific immune responses.

### 3.4 Neutralizing activity induced by co-administration of NiV-F and NiV-G mRNA-LNPs

**Figure 5:**
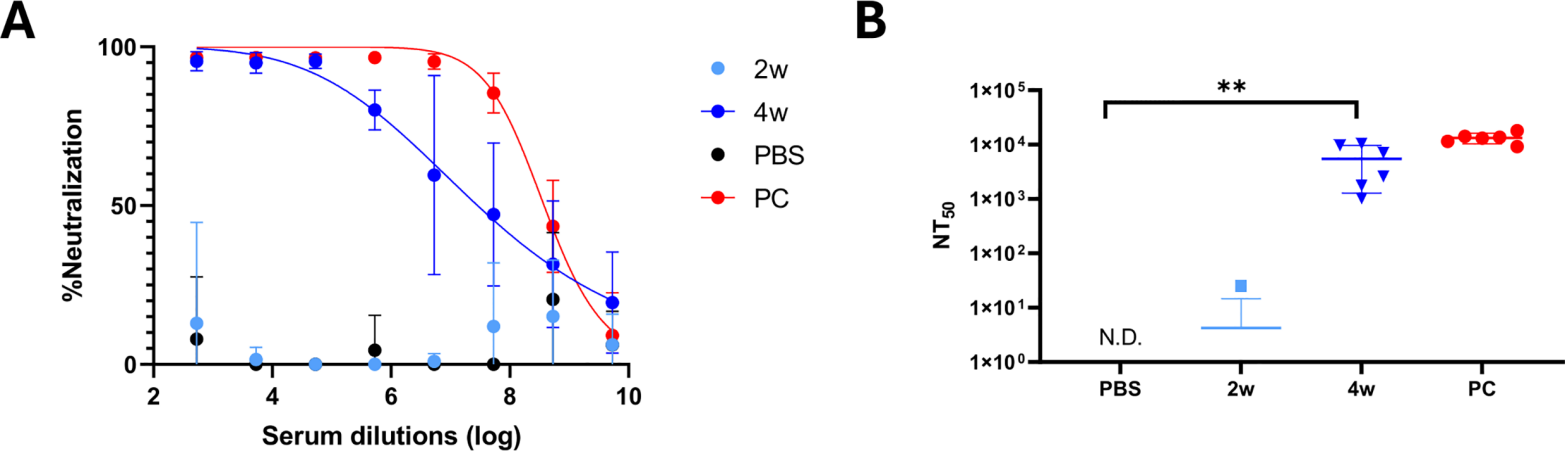
Neutralizing antibody responses against NiV pseudovirus. **(A)** Neutralizing activity of sera from the NiV mRNA-LNP–vaccinated group was evaluated using a pseudovirus based neutralization assay. Sera collected from NiV mRNA-LNP–vaccinated group and PBS control group, as well as commercially available polyclonal anti–NiV-G antibody (PC) were tested for neutralizing activity. The x-axis represents the log-transformed serial dilution of sera, ranging from 1:20 to 1:43,740 in three-fold dilutions. The y-axis indicates percent neutralization, calculated from RLU values measured after infection of target cells with virus–serum mixtures. **(B)** NT_50_ were calculated based on the neutralization curves shown in **(A)**. NT_50_ values were determined using GraphPad Prism. Individual data points represent NT_50_ values from individual mice. Statistical analysis was performed using ordinary one-way ANOVA (**P< 0.01).

To evaluate neutralizing antibody responses induced by co-administered NiV mRNA-LNP vaccines, a pseudovirus-based neutralization assay was performed (**Figures 5A, B**). Sera collected at week 2 post-immunization showed little to no neutralizing activity across the entire dilution range and exhibited levels comparable to those of the PBS control group. In contrast, sera collected at week 4 post-immunization displayed clear dose-dependent neutralizing activity (**Figure 5A**). Based on these results, neutralizing antibody titers were quantitatively assessed using NT_50_ values (**Figure 5B**). No neutralizing activity was detected in the PBS control group, and NT_50_ values were therefore denoted as not detected (N.D.). Similarly, sera collected at week 2 post-immunization showed no detectable neutralizing activity in most animals, with only a subset exhibiting very low NT_50_ values at approximately 1 × 10¹. In contrast, sera collected at week 4 post-immunization showed a significant increase in NT_50_ values, reaching approximately 10³–10^4^ in all animals, confirming robust induction of neutralizing antibodies. A commercial polyclonal anti-NiV-G antibody used as a positive control exhibited the highest NT_50_ values (>10^4^) and served as a validation control demonstrating the appropriate performance of the assay.

## 4 Discussion

NiV is a highly pathogenic zoonotic virus with recurrent outbreaks and limited therapeutic options, and no licensed vaccines are currently available. Given the constraints imposed by its BSL-4 classification, mRNA-based vaccine platforms offer a promising strategy for rapid and flexible vaccine development against NiV (18). Conventional methods of vaccine development need propagation or handling of infectious virus, while mRNA vaccines require only the genetic sequence encoding the antigen thereby, reducing biosafety risks (19). In this study, we designed NiV-F and NiV-G mRNA-LNP vaccine platform and applied a co-administration strategy, verified the quality of the synthesized mRNA and LNP formulations, and evaluated antigen-binding and *in vitro* neutralizing antibody responses in a mouse model. Taken together, our results suggest that the NiV mRNA-LNP vaccine design developed in this study were successfully synthesized and formulated into LNPs and were capable of inducing antigen-binding and neutralizing antibody responses.

The design of the NiV-F– and NiV-G–based mRNA vaccines incorporated structural stabilization strategies, including prefusion-stabilizing mutations in the F protein and the introduction of trimerization domains. Previous studies have reported that optimization of antigen structural design can enhance vaccine efficacy, and that stabilization of antigens in the prefusion conformation more effectively exposes key neutralizing epitopes, thereby inducing stronger immune responses (13). In addition, trimerization domains have been shown to promote the formation of native-like trimeric structures, enhance antigenicity and receptor binding (20). However, a limitation of this study is that structural validation confirming whether the introduced trimerization domains formed trimeric structures was not performed. Future studies employing structure-based analytical techniques, such as negative-stain electron microscopy, will be required to verify antigen oligomerization (13).

Quality assessment of the synthesized mRNA constructs suggested appropriate RNA quality and effective dsRNA removal, as confirmed by agarose gel electrophoresis and dot blot analysis. These results indicate that the NiV-F and NiV-G mRNA constructs were successfully synthesized with appropriate quality. It has been reported that the translational efficiency and stability of mRNA vaccines depend on mRNA structural integrity and the removal of impurities (21), and that residual dsRNA can significantly affect *in vivo* mRNA translation and stability (22). Accordingly, the stable mRNA synthesis and dsRNA removal strategy employed in this study is consistent with previous reports and meets fundamental quality requirements for mRNA vaccine platforms.

NiV is classified as a BSL-4 pathogen. Therefore, an important objective of this study was to establish a NiV pseudovirus system that enables assessment of neutralizing antibody under BSL-2 conditions (16). Consistent with previous reports indicating low pseudotyping efficiency of non-optimized lentiviral systems (14), the lentivirus-based pseudovirus approach yielded little detectable infectivity. In contrast, the VSV-*Δ*G-Luc–based pseudovirus system exhibited reproducible infectivity and markedly higher luciferase signals relative to baseline, in agreement with prior studies demonstrating the suitability of VSV-based systems for pseudotyping a wide range of enveloped viral glycoproteins (23). Meanwhile, *Δ*G-VSV-Luc lacks the VSV-G gene and therefore cannot produce infectious viruses in the absence of envelope protein expression. Therefore, infectious virus should theoretically not be present in the supernatant obtained from cells that do not express any viral envelope protein (24). However, we observed unexpectedly high background infectivity in supernatants derived from cells that did not express any viral envelope proteins. To address this issue, we optimized the washing steps and applied VSV-G antibody treatment, which substantially reduced this nonspecific background infectivity. These findings highlight an important technical consideration in VSV-based pseudovirus production and are consistent with previous observations (14). Nevertheless, the purification procedures required to suppress background infectivity may also reduce viral yield, representing a limitation of the current system. Future studies focusing optimization strategies may help to improve pseudovirus system while maintaining specificity (16). In addition, further validation using specificity controls such as Bald-VSV, VSV-G pseudotypes, single-antigen NiV-F or NiV-G conditions, and receptor-blocking approaches targeting ephrin-B2/B3 may help to more rigorously evaluate the specificity and quantitative reliability of the pseudovirus system.

Immunogenicity analyses revealed that both the NiV mRNA-LNP vaccine group and the recombinant NiV protein vaccine group induced antigen-specific binding antibody responses against both NiV-F and NiV-G under co-administration conditions. These results demonstrate that the antigen combination strategy used in this study was capable of eliciting detectable immune responses *in vivo*. Since this study was designed to evaluate the co-administration of independently formulated NiV-F and NiV-G mRNA-LNP, only the combined vaccine group was included. Our findings support the feasibility and immunogenic potential of the co-administration strategy. Future studies incorporating single-antigen groups and varying antigen ratios may provide further insight into antigen-specific contributions and further expand the applicability of this strategy.

Furthermore, sera collected at week 4 from the NiV mRNA-LNP–vaccinated mice exhibited significant neutralizing activity *in vitro*, indicating that this vaccine strategy can induce functional neutralizing antibodies in addition to binding antibodies. However, because *in vivo* viral challenge experiments were not performed, protective efficacy against live virus infection could not be directly evaluated. Future studies employing *in vivo* challenge models, such as IVIS-based bioluminescence imaging, will be required to assess *in vivo* protective efficacy.

In summary, the mRNA-LNP vaccine developed in this study demonstrated reproducible formulation characteristics, induction of humoral immune responses, and the generation of neutralizing antibodies, highlighting its potential as a NiV vaccine strategy. Notably, unlike previous approaches that primarily integrate multiple antigens into a single construct, the single-antigen mRNA-LNP co-administration strategy employed in this study demonstrates the feasibility of applying independently formulated antigen delivery for NiV mRNA vaccine design. By formulating NiV-F and NiV-G as an independent mRNA-LNPs and administering them in combination, this approach enables flexible adjustment of antigen composition and dosing ratios. This strategy provides expandability for future vaccine design, including the incorporation of additional antigens and the development of multivalent vaccine formulations. In addition, further optimization of antigen expression level, immunization schedule, vaccine dose, and incorporation of adjuvant strategies may improve immunogenicity and contribute to the development of a more robust vaccine candidate (10, 25). In conclusion, the accumulation of such studies is expected to contribute to the development of mRNA vaccine platforms capable of responding to highly pathogenic Nipah virus with pandemic potential.

## Data Availability

The original contributions presented in the study are included in the article/**Supplementary Material**. Further inquiries can be directed to the corresponding author.

## Supplementary material

Supplementary Figure 1 Effect of purification on VSV-based Nipah pseudovirus background signal.

Supplementary Figure 2 Full blot images corresponding to **Figure 2A**.

Supplementary Figure 3 NT_50_ values of the recombinant protein-immunized group.
