# Supplementary figures and images for: Development of Nipah virus mRNA vaccine for pandemic preparedness
Source: Front Immunol. 2026 Jun 5;17:1843559. doi: 10.3389/fimmu.2026.1843559 (PMC13279515; doi:10.3389/fimmu.2026.1843559)

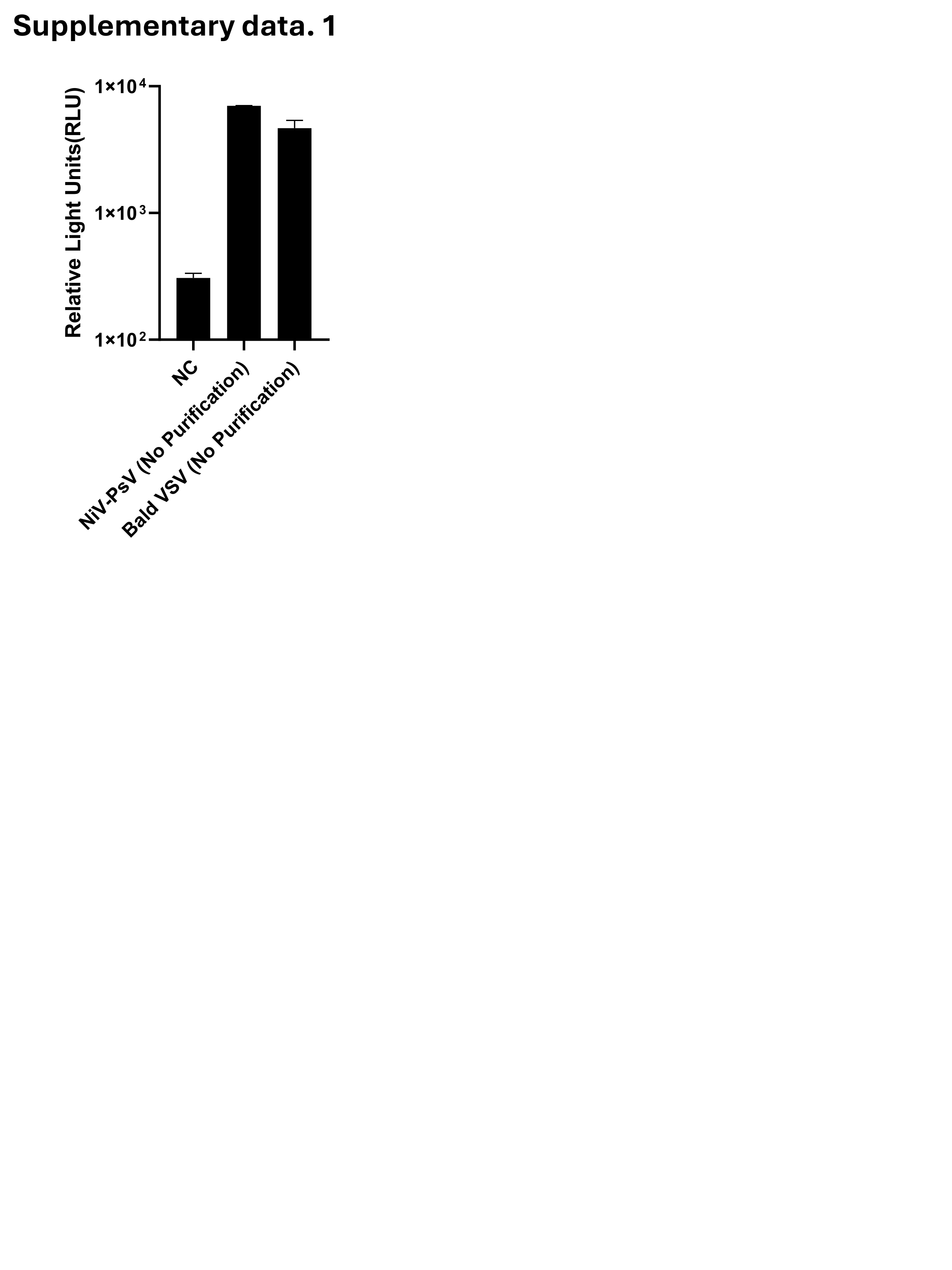

Supplement: Supplementary Figure 1 — Effect of purification on VSV-based Nipah pseudovirus background signal. [file Image1.tif]

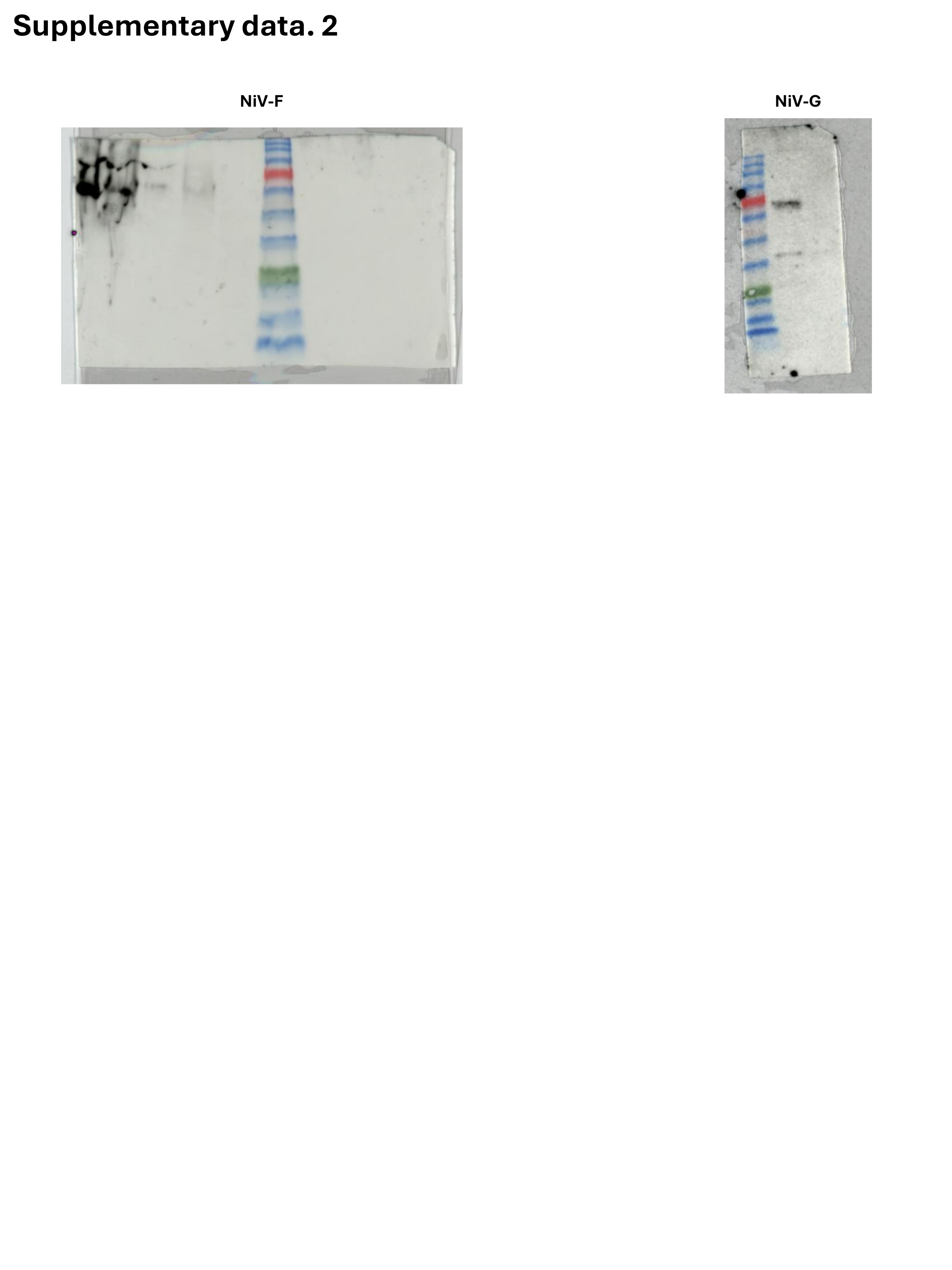

Supplement: Supplementary Figure 2 — Full blot images corresponding to Figure 2A. [file Image2.tif]

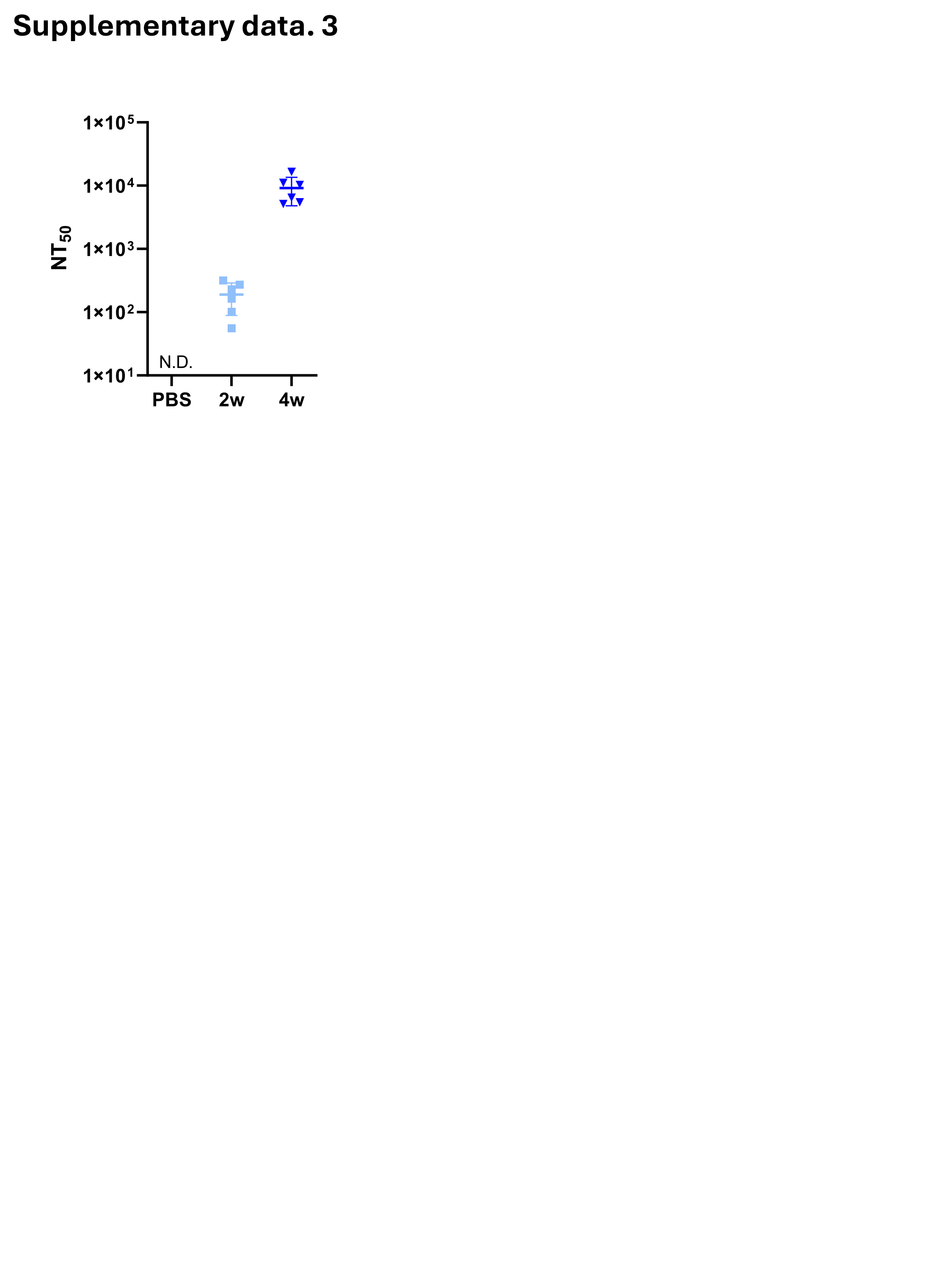

Supplement: Supplementary Figure 3 — NT50 values of the recombinant protein-immunized group. [file Image3.tif]
